# Impairment of glyoxalase-1, an advanced glycation end-product detoxifying enzyme, induced by inflammation in age-related osteoarthritis

**DOI:** 10.1186/s13075-018-1801-y

**Published:** 2019-01-11

**Authors:** Sabine Trellu, Alice Courties, Stéphane Jaisson, Laëtitia Gorisse, Philippe Gillery, Saadia Kerdine-Römer, Carlos Vaamonde-Garcia, Xavier Houard, François-Paul Ekhirch, Alain Sautet, Bertrand Friguet, Claire Jacques, Francis Berenbaum, Jérémie Sellam

**Affiliations:** 1 Sorbonne University, UPMC Univ Paris 06, Paris, France; 2 INSERM UMRS_938, CRSA, Paris, France; 3 Inflammation-Immunopathology-Biotherapy Department (DHU i2B), Paris, France; 4 Department of Rheumatology, Assistance Publique - Hôpitaux de Paris (AP-HP), Saint-Antoine Hospital, 184 rue du Faubourg Saint-Antoine, 75012 Paris, France; 5 UMR MEDyC CNRS/URCA 7369, University of Reims Champagne-Ardenne, Reims, France; 6 INSERM UMR 996, Univ Paris-Sud, University Paris-Saclay, Châtenay-Malabry, France; 7 Department of Physiotherapy, Cell Therapy and Regenerative Medicine Group, Medicine and Biological Science. Faculty of Health Sciences, University of A Coruña, 15006 A Coruña, Spain; 8 Groupe Maussins, Clinique des Maussins-Ramsay, Générale de Santé, Paris, France; 9 Department of Orthopedic Surgery, AP-HP, Saint-Antoine Hospital, Paris, France; 10 UMR 8256 - IBPS, CNRS UMR 8256, INSERM U1164, F-75005 Paris, France

**Keywords:** Aging, Advanced glycation end-product, Chondrocyte, Osteoarthritis, Glyoxalase, Carboxymethyl-lysine

## Abstract

**Background:**

Accumulation of advanced glycation end-products (AGEs) is involved in age-related osteoarthritis (OA). Glyoxalase (Glo)-1 is the main enzyme involved in the removal of AGE precursors, especially carboxymethyl-lysine (CML). We aimed to investigate the expression of several AGEs and Glo-1 in human OA cartilage and to study chondrocytic Glo-1 regulation by inflammation, mediated by interleukin (IL)-1β.

**Methods:**

Ex vivo, we quantified AGEs (pentosidine, CML, methylglyoxal-hydroimidazolone-1) in knee cartilage from 30 OA patients. Explants were also incubated with and without IL-1β, and we assessed Glo-1 protein expression and enzymatic activity. In vitro, primary cultured murine chondrocytes were stimulated with increasing concentrations of IL-1β to assess Glo-1 enzymatic activity and expression. To investigate the role of oxidative stress in the IL-1β effect, cells were also treated with inhibitors of mitochondrial oxidative stress or nitric oxide synthase.

**Results:**

Ex vivo, only the human cartilage CML content was correlated with patient age (*r* = 0.78, *p* = 0.0031). No statistically significant correlation was found between Glo-1 protein expression and enzymatic activity in human cartilage and patient age. We observed that cartilage explant stimulation with IL-1β decreased Glo-1 protein expression and enzymatic activity. In vitro, we observed a dose-dependent decrease in Glo-1 mRNA, protein quantity, and enzymatic activity in response to IL-1β in murine chondrocytes. Inhibitors of oxidative stress blunted this downregulation.

**Conclusion:**

Glo-1 is impaired by inflammation mediated by IL-1β in chondrocytes through oxidative stress pathways and may explain age-dependent accumulation of the AGE CML in OA cartilage.

**Electronic supplementary material:**

The online version of this article (10.1186/s13075-018-1801-y) contains supplementary material, which is available to authorized users.

## Background

Osteoarthritis (OA) is no longer considered a unique disease [1], and we currently divide OA into several phenotypes based on the main risk factors involved. Likewise, we designate OA as post-traumatic OA, age-related OA, and metabolic syndrome (MetS)-associated OA [2]. These phenotypes may display specific pathophysiological pathways and require specific treatments.

Although MetS-associated OA has been extensively investigated [3], the mechanisms linking age and OA pathogenesis are still not completely understood. Cellular senescence and extracellular matrix alterations are known to be involved in the age-related OA phenotype [4, 5], but the accumulation of advanced glycation end-products (AGEs) and other post-translational-modified proteins [6] is also one of the key features of the OA cartilage due to aging or metabolic processes; however, AGE accumulation has been poorly studied in the context of OA [7]. These products are formed by successive nonenzymatic reactions between a sugar and a protein, an amino acid, or a lipid [8]. Several types of AGE are generated by these reactions. Quantitatively, the main AGEs are hydro-imidazolones, such as methylglyoxal-hydroimidazolone-1 (MG-H1), but N_ε_-carboxymethyl-lysine (CML), N_ε_-carboxyethyl-lysine (CEL), and pentosidine have also been reported [8]. AGE formation increases in some pathological conditions, such as diabetes mellitus [9]. Importantly, AGE formation is irreversible, leading to tissue accumulation and ultimately irreversible tissue damage. AGE accumulates in the collagen network of healthy cartilage with aging because of its low regenerative capacity [7]. AGEs affect cartilage biomechanical properties by increasing the stiffness of the collagen network [10], inhibit type II collagen synthesis [11], disturb the activity of metalloproteinases [12], and have proinflammatory and pro-oxidative effects on chondrocytes by binding to their receptor (RAGE) [13]. Some serum glycation markers, such as glucosepane, are also correlated with OA in vivo [14]. However, despite the demonstrated role of AGE accumulation in cartilage aging, the chemical mechanisms leading to AGE synthesis and accumulation have been poorly assessed in OA cartilage [15–17].

To limit AGE accumulation in tissues, detoxification mechanisms eliminate AGE precursors (i.e., glyoxal and methylglyoxal) which involves the glyoxalase enzymatic system, including glyoxalase (Glo)-1 and Glo-2. These mechanisms convert methylglyoxal/glyoxal to d-lactate/glycolate via S-d-lactoylglutathione/S-2-hydroxyethyglutathione, respectively [18, 19]. The reduced glutathione (GSH) serves as a cofactor and is regenerated during the process. In this system, the main and limiting enzyme is Glo-1, which is ubiquitous, cytosolic, and active in its dimeric form [19, 20]. With aging, Glo-1 mRNA and protein expression and enzymatic activity decrease in the brain and red blood cells [19] but increase in other tissues such as the skin [21]. Interestingly, Glo-1 overexpression can increase longevity of worms [22]. Glo-1 restoration is currently under investigation for the prevention of aging-related disorders [23]. To date, involvement of Glo-1 in OA has only been speculated since OA is an AGE-related disease model [12] and because Glo-1 is involved in aging diseases [20, 24].

Chronic inflammation may also be involved in cartilage aging. Indeed, with age, chronic sterile low-grade inflammation, called “inflammaging”, manifests [25] and is locally amplified by the senescence-associated secretory phenotype (SASP) of chondrocytes [26]. This phenomenon is involved in several age-related diseases, such as cardiovascular or neurodegenerative diseases, as well as cancer [27, 28]. Interestingly, in the elderly, the interleukin (IL)-6 serum concentration, which is associated with the SASP, may predict 5-year mortality [29]. Inflammaging may also be involved in chondrosenescence and therefore age-related OA [30–32]. Indeed, increased levels of inflammatory markers such as serum IL-6, C-reactive protein, or bioactive lipids are associated with knee OA progression [33–35], illustrating the role of chronic low-grade inflammation in OA. Moreover, the biological behavior of OA chondrocytes may be influenced by a patient’s age; IL-1β-stimulated human OA chondrocytes show increased release of matrix metalloproteinase-13 with increasing age of the cartilage donor [36].

Inflammation also induces oxidative stress in chondrocytes through the production of nitric oxide (NO) and mitochondrial reactive oxygen species (ROS) [37]. An increasing intracellular ROS rate stimulates nuclear factor-erythroid 2-related factor-2 (Nrf-2), which is a transcription factor involved in the cellular response to oxidative stress [38]. The Glo-1 promotor has a binding domain for Nrf-2 and can thus be upregulated in an inflammatory and pro-oxidative context [39].

Here, we aimed to investigate the extracellular matrix AGE content and chondrocytic Glo-1 expression in OA cartilage along with the study of Glo-1 regulation by oxidative stress.

## Methods

### Collection of human OA cartilage

Human OA knee explants were obtained from patients undergoing total knee arthroplasty due to OA at Saint-Antoine Hospital (Paris) or at the Maussins clinic (Paris) shortly after surgery (BioJOINT, which is a biobank of human knee OA, legal authorization: CPP Paris Ile de France V, CNIL reference: MMS/HGT/AR177404). Informed consent for the use of tissue and clinical data was obtained from each patient before surgery. Experiments with human samples were approved by a French Institutional Review Board (Comité de Protection des Personnes, Paris Ile de France 5 and Commission Nationale de l’Informatique et des Libertés). For each explant, a biopsy of the cartilage was performed, which was then embedded in paraffin. All remaining cartilage zones were manually dissected (i.e., tibial plateaus and femoral condyles), cut into small pieces (≈ 5 mm^3^), and mixed to obtain homogenous isolated cartilage samples. The cartilage pieces were washed with phosphate-buffered saline (PBS) and incubated in Dulbecco’s modified Eagle’s medium (DMEM) containing 25 mM glucose, which is necessary for human explant maintenance, and supplemented with 100 U/mL penicillin (P), 100 mg/mL streptomycin (S), and 4 mM glutamine (Glu) for 24 h at 37 °C, with or without IL-1β (5 ng/mL) (Peprotech, Rocky Hill, NJ, USA), as described previously [37]. Each volume of medium was normalized to the wet weight of the explants (6 mL/g tissue). The incubated explants were then collected, centrifuged (1600 g for 6 min), the medium was discarded, and the cartilage explant was stored at −20 °C for later analysis. The AGE products were not measured in the explant medium because the explants were incubated for only 24 h. This time duration is too short to observe relevant AGE release [40]. Moreover, to be closer to what happens in vivo in human OA, we preferred to quantify AGE directly within the cartilage explants instead of measuring AGE in the media.

### Primary culture of murine articular chondrocytes

Mouse primary chondrocytes were isolated from the articular cartilage of 5- to 6-day-old C57Bl6 mice from Janvier (St. Berthevin, France) and seeded at 8 × 10^3^ cells per cm^2^ as previously described [41]. Articular chondrocytes obtained from newborn mice using this protocol are considered appropriate to study mature chondrocytes since type II collagen and aggrecan mRNAs are highly expressed, while the expression of type I collagen mRNA remains low in these cells [42]. After 1 week of amplification in DMEM (5.5 mM glucose) P/S/Glu supplemented with 10% fetal calf serum, the cells were incubated in serum-free DMEM P/S/Glu containing 0.1% bovine serum albumin for 24 h before treatment (basal experimental medium). Each littermate of the mice was used for one experiment. All experiments with murine articular chondrocytes were performed according to protocols approved by the French and European ethics committees (Comité Régional d’Ethique en Expérimentation Animale N°3 de la région Ile de France).

### Treatment of primary cultures of murine chondrocytes

After 24 h of incubation in basal experimental medium, murine chondrocytes were stimulated for 72 h with increasing IL-1β concentrations (0 to 10 ng/mL). Cell lysates were collected for mRNA and protein extractions after 72 h. For mechanistic studies investigating the roles of ROS and NO in Glo-1 regulation, chondrocytes cultured for 72 h with or without IL-1β (5 ng/mL) were coincubated with inhibitors for 72 h: MitoTEMPO (50 mM; Santa Cruz Biotechnology, Heidelberg, Germany), a specific scavenger of mitochondrial ROS, or l-NAME (5 mM; Sigma Aldrich, Saint-Louis, MO, USA), a nonspecific inhibitor of NO synthase. The doses of these inhibitors were selected according to data in the literature and had been previously used in similar experiments [37]. In addition, experiments were also performed using Nrf-2 knockout (Nrf-2^−/−^) mice generated from inbred Nrf-2 heterozygous mice on a C57BL/6 J background, as previously described [43]. The mice were housed in a pathogen-free facility and handled in accordance with the principles and procedures outlined in Council Directive 86/609/EEC. Nrf-2^−/−^ chondrocytes were cultured for 72 h with or without IL-1β (5 ng/mL) as IL-1β is able to induce Nrf-2 in wild-type animals [44]. All measurements were performed in duplicate, and the mean of the duplicates from one littermate of the mice was considered one experiment for each condition.

### CML, pentosidine, and MG-H1 quantification

Quantification of CML, pentosidine, and MG-H1 in human cartilage was performed by liquid chromatography coupled with tandem mass spectrometry (LC-MS/MS). Briefly, human cartilage (∼ 100 mg) was homogenized with 1 mL 0.5 M acetic acid in Lysing Matrix D Tubes with the FastPrep-24 System (MP Biomedicals). After homogenization, the samples were digested with pepsin (10% w/w) for 24 h at 37 °C. They were then subjected to acid hydrolysis in 6 M hydrochloric acid for 18 h at 110 °C. Hydrolysates were evaporated and resuspended in 125 mM ammonium formate before quantification by LC-MS/MS. CML and pentosidine were assayed by LC-MS/MS (API4000 system ABSciex, Les Ulis, France) as previously described [45, 46]. For MG-H1 measurements, dried hydrolysates resuspended in 100 μL of 125 mM ammonium formate containing 20 μM d_3_-MG-H1 (used as an internal standard) were filtered using Uptidisc PTFE filters (4 mm, 0.45 μm; Interchim, France) prior to LC-MS/MS analysis. Chromatographic separation was performed using a Kinetex PFP column (100 × 4.6 mm, 2.6 μm; Phenomenex) with a gradient program composed of 5 mM ammonium formate (pH 2.9) as mobile phase A and 100% acetonitrile as mobile phase B. The flow rate was constant at 0.7 mL/min during all separation steps. The gradient program was as follows: 0–0.5 min: 0% B; 0.5–2.2 min: gradient to 40% B; 2.2–2.6 min: gradient to 95% B; 2.6–3.6 min: 95% B; 3.6–3.7 min: gradient to 0% B; and 3.7–6.0 min: 0% B. The injection volume was 4 μL, and the oven temperature was set at 30 °C. Detection was performed using an API4000 system in positive-ion mode with an electrospray ionization (ESI) source. Multiple reaction monitoring transitions were as follows: 229.2 > 166.1 and 229.2 > 212.2 for the quantification and confirmation transitions, in contrast to 232.2 > 169.1 for the internal standard. For all AGEs, the results are expressed as ratios relative to lysine content.

### RNA extraction and quantitative RT-PCR (RT-qPCR)

Total RNA was extracted from murine chondrocytes using the ReliaPrep RNA Cell Miniprep System kit (Promega, Madison, WI, USA), and concentrations were determined by spectrophotometry (Eppendorf, Le Pecq, France). Reverse transcription was performed with 500 ng of total RNA with the Omniscript RT kit (Qiagen). Glo-1 mRNA levels were quantified with the Light Cycler LC480 (Roche Diagnostics, Indianapolis, IN, USA). PCR amplification conditions were as follows: initial denaturation for 5 min at 95 °C, followed by 40 cycles consisting of 10 s at 95 °C, 15 s at 60 °C, and 10 s at 72 °C. Product formation was detected at 72 °C in the fluorescein isothiocyanate channel. The mRNA levels were normalized to those of murine hypoxanthine guanine phosphoribosyltransferase (HPRT). Specific mouse primer sequences were as follows: Glo-1, forward 5′- CCTGATGACGGGAAAATGAAAG -3′, and reverse 5’-GCCGTCAGGGTCTTGAATGA-3′; HPRT, forward 5′- AGGACCTCTCGAAGTGT-3′, and reverse 5’-ATTCAAATCCCTGAAGTACTCAT-3′. All measurements were performed in duplicate.

### Protein extraction and Western blot analysis

Total proteins were extracted from murine chondrocytes using lysis buffer (RIPA buffer containing a protease inhibitor cocktail; Roche Diagnostics, Indianapolis, IN, USA). Human OA explants incubated in DMEM with or without IL-1β were frozen in liquid nitrogen and then manually crushed. Homogenates were incubated in lysis buffer with agitation for 1 h at 4 °C and then centrifuged at 13,000 rpm for 1 h at 4 °C. Proteins were collected from supernatants and quantified by absorbance with the BCA protein assay kit (Thermo Scientific, Waltham, MA, USA).

For Western blot analysis, protein samples were diluted in Laemmli buffer, under nonreducing conditions, to retain the dimeric active form of Glo-1. Next, 14 μg and 13 μg of protein per well from murine and human samples, respectively, were loaded onto a 4–12% SDS polyacrylamide gel and separated by electrophoresis. The proteins were then transferred onto a nitrocellulose membrane.

The nitrocellulose membrane was blocked with 5% (m/v) milk diluted in Tris-buffered saline and Tween 20 (TBST) for 1 h, and then incubated overnight at 4 °C with the primary antibody, a monoclonal rabbit anti-Glo-1 antibody (diluted 1/200 in 5% (m/v) milk TBST; Santa Cruz Biotechnology, Heidelberg, Germany). After washing with TBST, the membrane was incubated with horseradish peroxidase-conjugated anti-rabbit antibody (diluted 1/1000 in 5% milk TBST) for 1 h at room temperature, and then submitted for detection using clarity Western ECL substrate (Bio-Rad, Hercules, CA, USA). The absorbance of the dimeric band was quantified using Imagelab software (Bio-Rad, Hercules, CA, USA) and normalized by global Ponceau staining quantification.

### Glo-1 enzymatic activity

To assess Glo-1 enzymatic activity, the formation of its product (S-d-lactoylglutathione) was measured by ultraviolet spectrophotometry using a specific assay (Sigma Aldrich, Saint-Louis, MO, USA). Per sample, the reagents were as follows: 8 μL of methylglyoxal, 8 μL of GSH, and 160 μL of phosphate buffer. The mixture was incubated for 5 min to ensure equilibration of hemithioacetal formation, the Glo-1 substrate, from methylglyoxal and GSH. Next, 10 μg of protein was added to each well in duplicate, and 160 μL of the reactive mixture was added to each well. The absorbance at 240 nm reflecting S-d-lactoylglutathione formation was measured at the beginning of the reaction and after 10 min of incubation at 25 °C. Enzymatic activity was calculated from the initial and final absorbances and expressed in units/mg protein. One unit is the amount of enzyme catalyzing the formation of 1 μmole of S-d-lactoylglutathione per minute.

### Fluorescence immunohistochemistry

Glo-1 protein expression was assessed in human OA cartilage by fluorescence immunochemistry. Samples were deparaffinized in three xylene baths for 5 min each and then rehydrated in baths containing decreasing alcohol concentrations and distilled water. Enzymatic unmasking was performed with hyaluronidase and pronase. H_2_O_2_ was applied for 30 min to inhibit endogenous peroxidase. Monoclonal rabbit anti-Glo-1 antibody (dilution 1/50; Santa Cruz Biotechnology, Heidelberg, Germany) was added to the samples, followed by incubation overnight at 4 °C. The negatives controls were dilution buffer and rabbit immunoglobulins (concentration 20 g/L, diluted 1/50). Secondary antibody (fluorophore horseradish peroxidase-conjugated anti-rabbit antibody, diluted 1/100) was incubated with the samples for 30 min at room temperature. The signal was then amplified by tyramide signal amplification. Nuclei were stained with DAPI.

### Statistical analysis

All tests were nonparametric and were performed with GraphPad Prism 7 (GraphPad Software, San Diego, CA, USA). The Wilcoxon paired test was used for all mice and human experiments. Spearman’s correlation was used to analyze any correlation between two continuous variables. *P* < 0.05 was considered statistically significant. All data are reported as the median and interquartile range.

## Results

### Description of the population

For all human experiments, we used 30 knee cartilage explants from OA patients. Due to the availability of the samples, two sets of patients were used for AGE and Glo-1 experiments, respectively, and only two patients underwent both experiments. The mean age was 67.4 ± 7.9 years, 82% of the patients were women, and the mean body mass index (BMI) was 29.7 ± 4.5 kg/m^2^ in the population assessed for AGE experiments. In the population for Glo-1 experiments, the mean age was 69.7 ± 9 years, 76% were women, and the mean BMI was 28.8 ± 5 kg/m^2^. Diabetic patients were excluded to avoid bias concerning CML or Glo-1 assessment. No significant difference was found between the two sets of data. The patients’ clinical characteristics are further described in Additional file 1 (Table S1).

### CML accumulates in human OA cartilage with aging

**Fig. 1:**
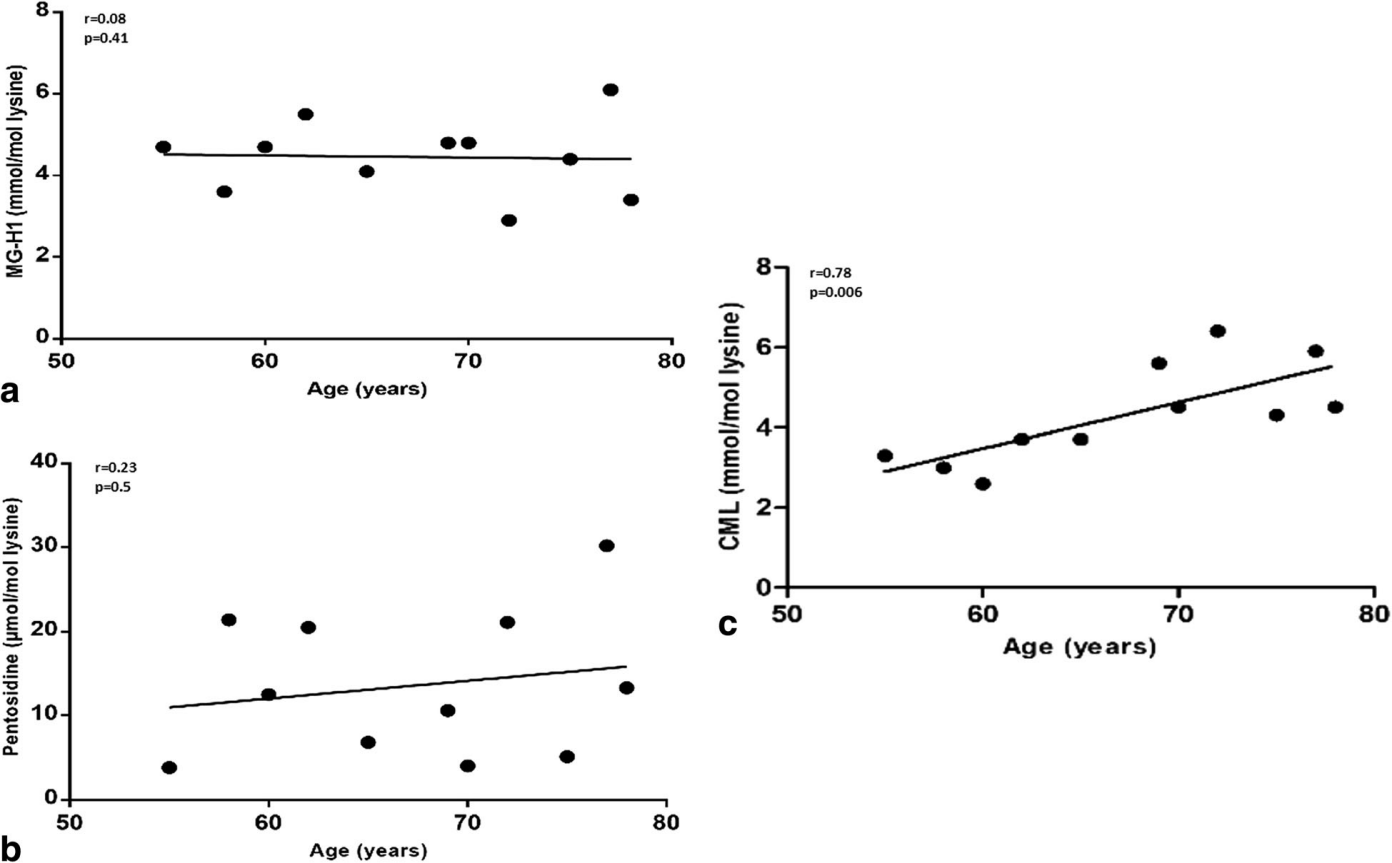
Correlations between pentosidine, carboxymethyl-lysine (CML), and methylglyoxal-hydroimidazolone-1 (MG-H1) concentrations and patient age in human osteoarthritic cartilage. **a** MG-H1 quantified by liquid chromatography coupled to mass spectrometry. **b** Pentosidine quantified by liquid chromatography coupled to mass spectrometry. **c** CML quantified by liquid chromatography coupled to mass spectrometry. *n* = 11 in each experiment

To evaluate the glycation status of the human OA cartilage, we quantified several AGEs such as CML, MG-H1, and pentosidine in cartilage total extracts. While no correlation was found between MG-H1 or pentosidine levels and patient age (Fig. 1), the CML concentration in OA cartilage was highly correlated with patient age (*r* = 0.78, *p* = 0.0031; Fig. 1). As a cofounding factor, the relationship between BMI and AGE concentration was studied. A negative correlation was found between BMI and CML (*r* = −0.7, *p* = 0.04; data not shown), and no correlation was found between MG-H1 or pentosidine levels and BMI. As no correlation was found between patient age and BMI, we consider these two variables to be independent. The relationship between CML concentration and cartilage histological damage or progression of the disease was not studied as each explant was obtained from a patient at the arthroplasty stage.

### No correlation between Glo-1 and patient age

To determine whether CML accumulation in human OA cartilage was related to Glo-1 disturbance, we first evaluated Glo-1 expression in the OA cartilage explants by immunofluorescence. Three randomly selected OA cartilage samples were analyzed, and Glo-1 was detected in the cytoplasm of chondrocytes in all samples (Additional file 1: Figure S1).

**Fig. 2:**
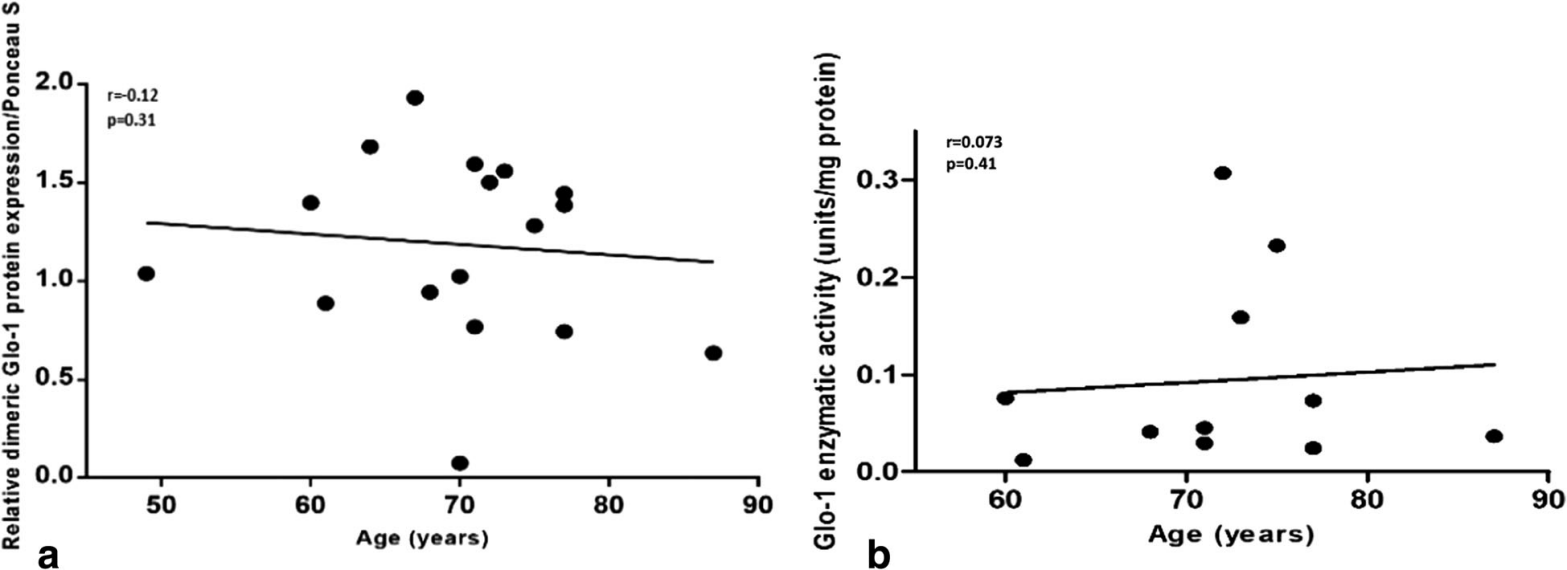
Relationships between glyoxalase-1 (Glo-1) protein expression and enzymatic activity and age in human OA cartilage. **a** Relationship between Glo-1 protein expression in human OA cartilage and patient age, *n* = 18. Two data points were similar and therefore are not visually separated. **b** Relationship between Glo-1 enzymatic activity in human OA cartilage and patient age. One unit is one S-d-lactoylglutathione micromole formed in 1 min, *n* = 11

Glo-1 protein expression and enzymatic activity were then assessed in human OA cartilage. All samples used in the Glo-1 enzymatic experiments were also used in the Glo-1 protein expression experiments. The three final samples were analyzed by immunofluorescence but were not available for the others experiments. Conversely to CML, Glo-1 protein expression did not change according to the age of the donor (Fig. 2a). Similarly, no statistically significant correlation was found between Glo-1 enzymatic activity and the age of the donor (Fig. 2b). In OA cartilage, CML accumulates with patient age, but Glo-1 seems to be unregulated by age and thus does not increase to limit this accumulation. We hypothesized that Glo-1 impairment could be due to inflammation occurring in age-related OA. After obtaining results in cartilage explants, that are closer to human OA than human OA chondrocytes, we have investigated mechanistically how IL-1β regulates Glo-1 using murine chondrocytes [40]. Primary culture of chondrocytes has been previously validated as relevant for exploring the intracellular and molecular features of chondrocyte activation [40].

### IL-1β decreases Glo-1 protein expression and enzymatic activity in cartilage

**Fig. 3:**
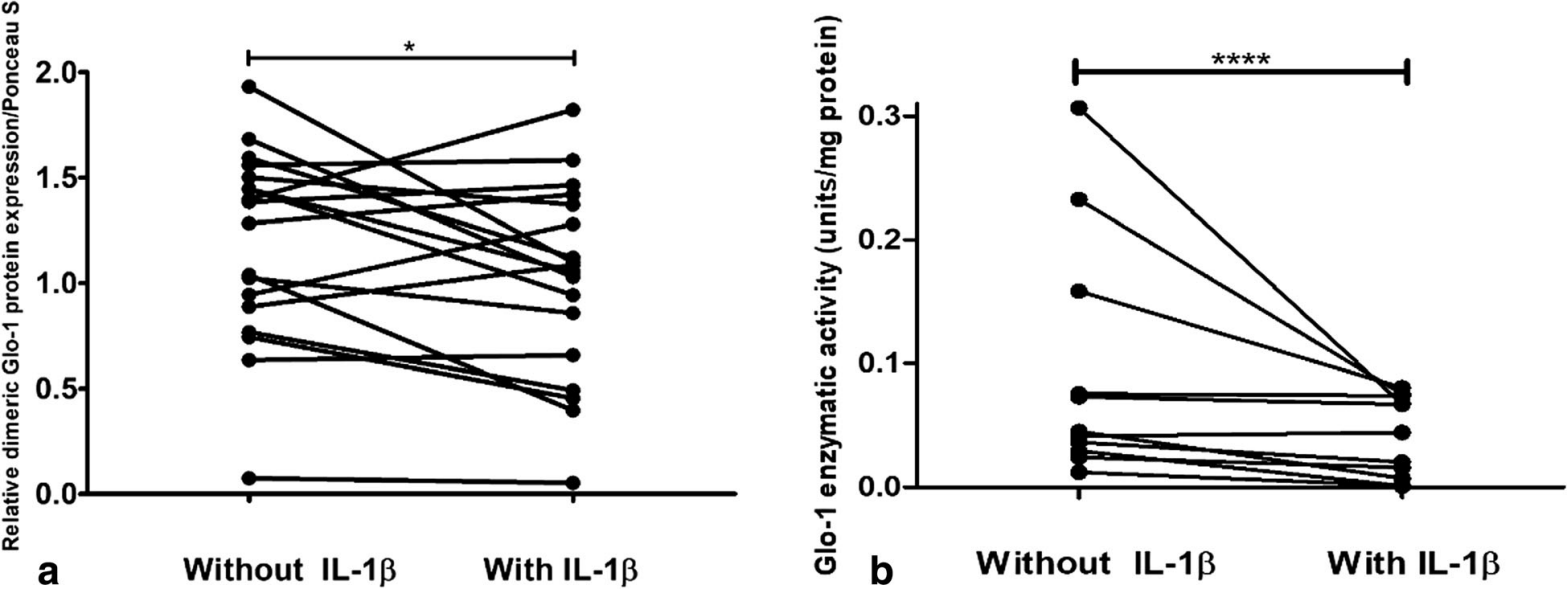
interleukin-1β (IL-1β) decreases glyoxalase-1 (Glo-1) protein expression and enzymatic activity in human OA cartilage. **a** Glo-1 protein expression in human OA cartilage from the same patient with or without IL-1β stimulation for 24 h, *n* = 18 in each group. **b** Glo-1 enzymatic activity in human OA cartilage from the same patient with or without IL-1β stimulation, *n* = 11 in each group. *p = 0.049, ****p = 0.002

Stimulation of cartilage explants with IL-1β significantly decreased the Glo-1 protein expression (1.33 ± 0.58 vs 1.07 ± 0.64, *p* = 0.049, without and with IL-1β, respectively; Fig. 3a) and Glo-1 enzymatic activity (0.045 ± 0.084 vs 0.044 ± 0.059, *p* = 0.002, without and with IL-1β, respectively; Fig. 3b) in human OA cartilage. The decrease in Glo-1 enzymatic activity or protein expression induced by IL-1β in human OA cartilage explants was similar regardless of the age of the donor (i.e., no correlation with patient age, data not shown).

**Fig. 4:**
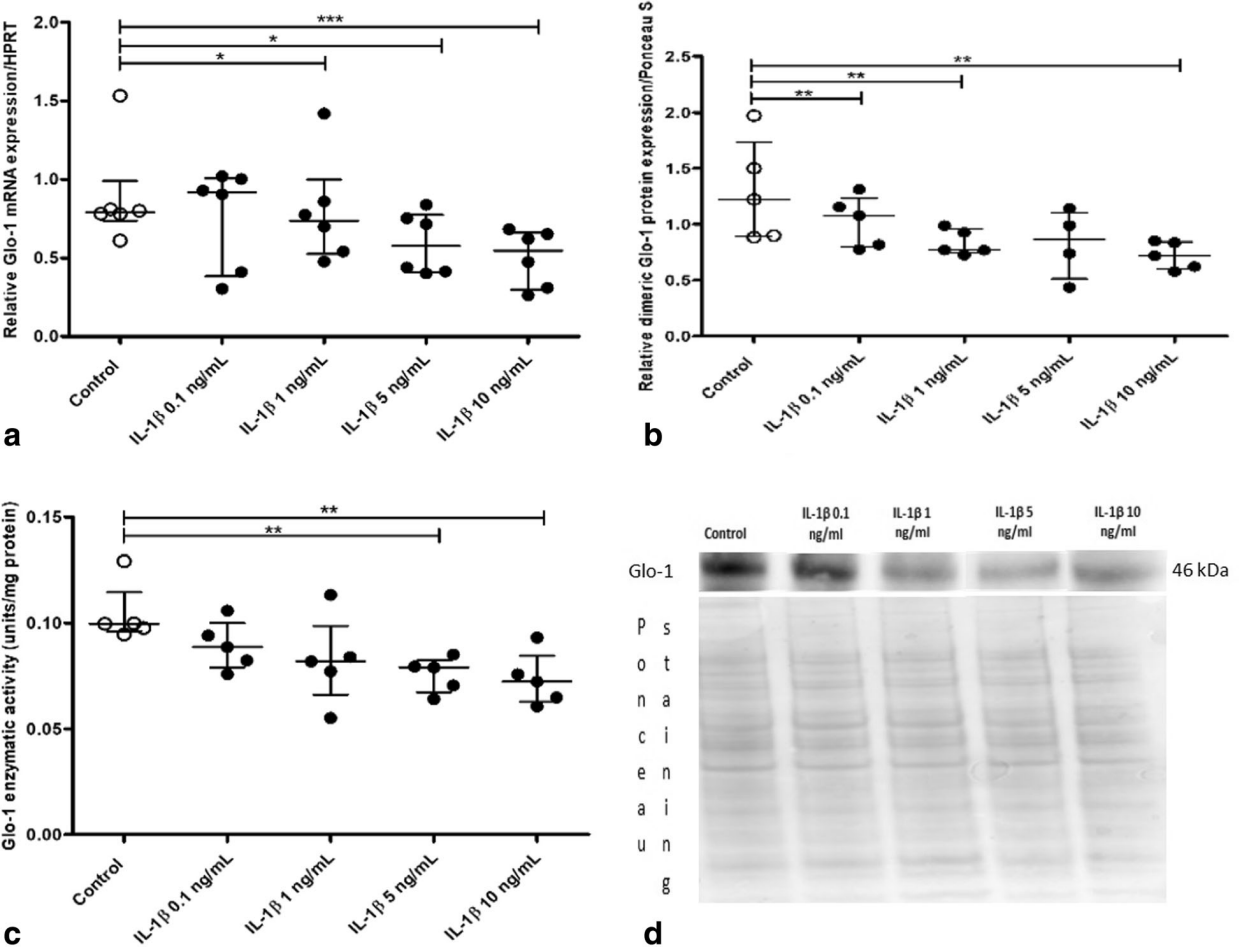
Glyoxalase-1 (Glo-1) expression or enzymatic activity in a primary culture of murine chondrocytes stimulated by increasing concentrations of interleukin-1β (IL-1β). **a** Quantitative RT-PCR analysis of mRNA levels of Glo-1 relative to hypoxanthine guanine phosphoribosyltransferase (HPRT), *n* = 6 for each condition. **b** Dimeric Glo-1 protein expression relative to Ponceau staining, *n* = 5 in each group. **c** Glo-1 enzymatic activity, *n* = 5 in each group. **d** Ponceau staining and Western blot as an illustrative example. Data are presented as median and interquartile range. *p = 0.0469, **p = 0.0313, ***p = 0.0156

To further explore the effect of IL-1β on Glo-1 expression, primary cultures of murine chondrocytes were subjected or not to IL-1β (0.1 to 10 ng/mL for 72 h), and Glo-1 expression was assessed. Similar to the results obtained with IL-1β-stimulated human OA cartilage explants, Glo-1 mRNA, protein expression, and enzymatic activity were decreased in response to IL-1β stimulation in a dose-dependent manner in culture murine chondrocytes (Fig. 4a–c). The highest IL-1β concentration (10 ng/mL) significantly decreased the Glo-1 mRNA level (0.69-fold, *p* = 0.02), dimeric protein expression (0.59-fold, *p* = 0.03), and enzymatic activity (0.72-fold, *p* = 0.03) compared with those in unstimulated cells.

### The decrease in Glo-1 induced by IL-1β is significantly limited by oxidative stress inhibition

**Fig. 5:**
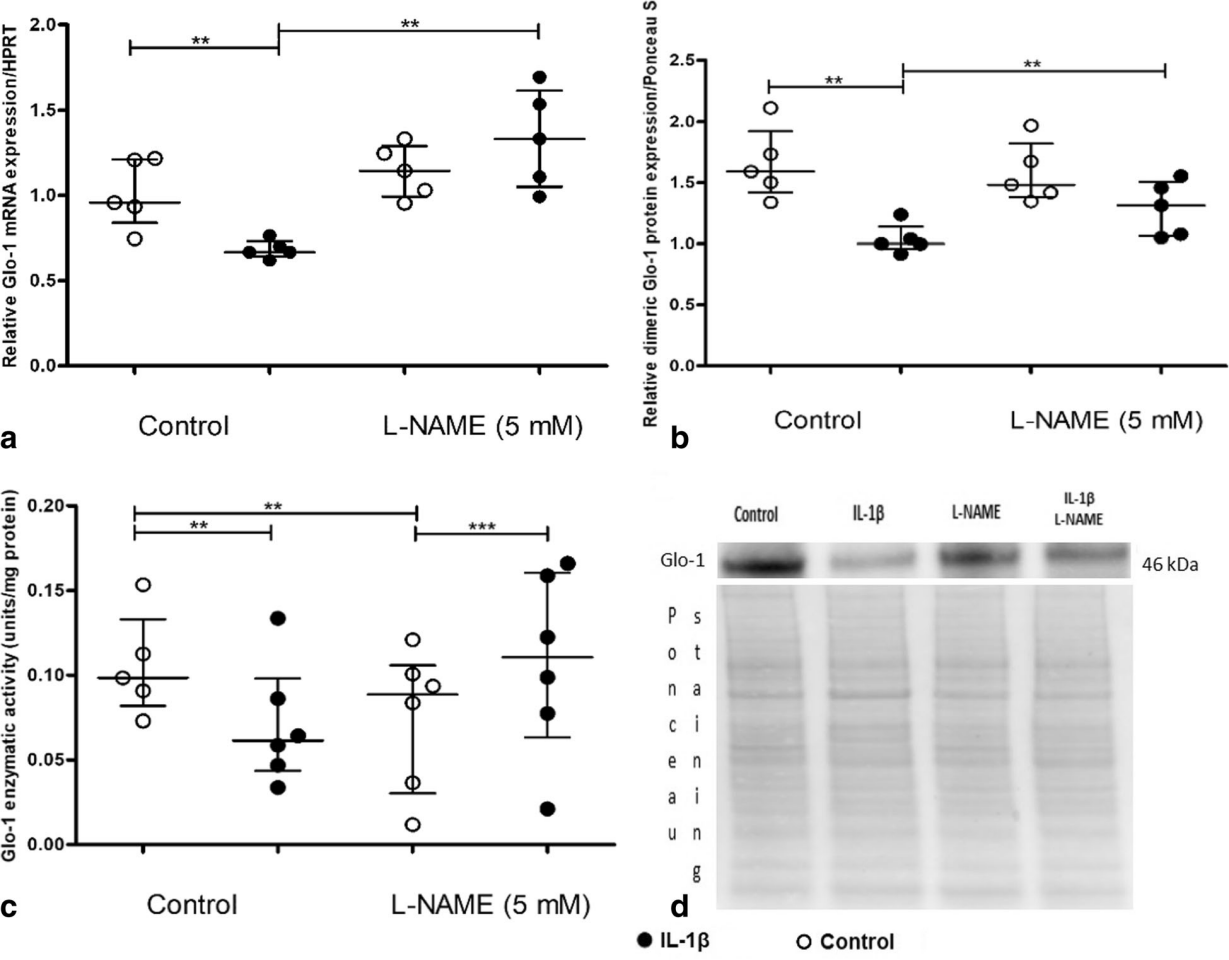
Involvement of nitric oxide (NO) in the downregulation of glyoxalase-1 (Glo-1) induced by interleukin-1β (IL-1β) in a primary culture of murine chondrocytes. Cells were treated with an inhibitor of NO synthase, l-NAME (5 mM). **a** Quantitative RT-PCR analysis of mRNA levels of Glo-1 relative to hypoxanthine guanine phosphoribosyltransferase (HPRT), *n* = 5 for each condition. **b** Dimeric Glo-1 protein expression relative to Ponceau staining, *n* = 5 in each group. **c** Glo-1 enzymatic activity, *n* = 6 in each group. **d** Ponceau staining and Western blot as an illustrative example. Data are presented as median and interquartile range. **p = 0.0313, ***p = 0.0156

Considering the relationship between oxidative stress and inflammation, we aimed to determine whether Glo-1 downregulation by IL-1β was mediated by oxidative stress using two inhibitors, l-NAME and MitoTEMPO, which block NO synthase and mitochondrial oxidative stress, respectively [34]. The IL-1β dose (5 ng/mL) was selected based on the plateau effect observed in protein expression and Glo-1 enzymatic activity with the 5-ng/mL IL-1β dose and the low cell toxicity at this IL-1β concentration (data not shown).

l-NAME prevented the decreased expression of Glo-1 mRNA (mean fold increase of 1.99 between IL-1β alone and IL-1β plus l-NAME, *p* = 0.03) and Glo-1 protein (mean fold increase of 1.31 between IL-1β alone and IL-1β plus l-NAME, *p* = 0.03), but it did not significantly affect enzymatic activity in IL-1β-stimulated chondrocytes (mean fold increase of 1.8 between IL-1β alone and IL-1β plus l-NAME, *p* = 0.08) (Fig. 5a–c). l-NAME alone seemed to have the same effect as IL-1β on Glo-1 enzymatic activity in chondrocytes (no difference was observed on incubation with IL-1β (median 0.061) or l-NAME (median 0.089), *p* = 0.42).

**Fig. 6:**
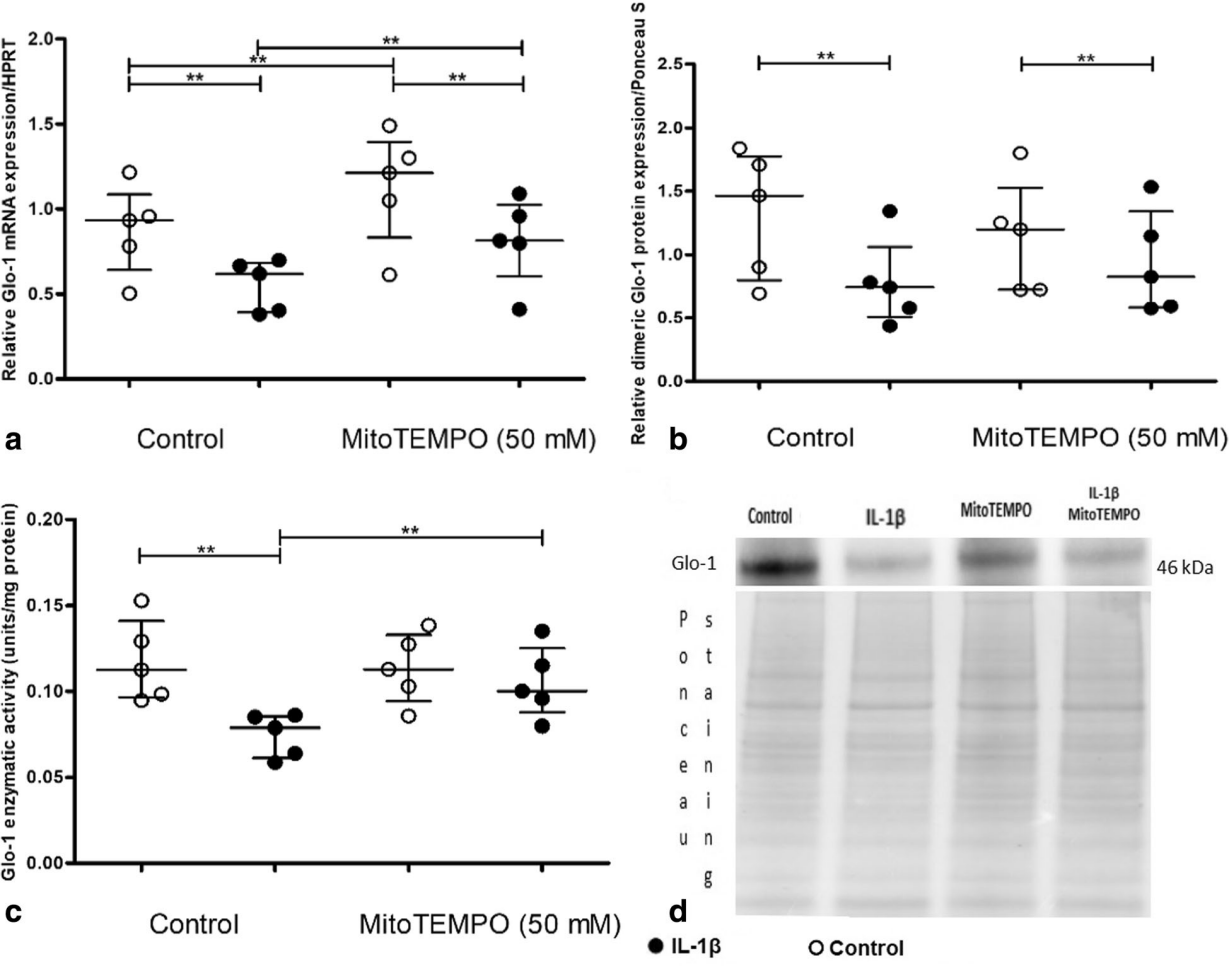
Involvement of mitochondrial oxidative stress in the downregulation of glyoxalase-1 (Glo-1) induced by interleukin-1β (IL-1β) in a primary culture of murine chondrocytes. Cells were treated with a scavenger of mitochondrial ROS, MitoTEMPO (50 mM). **a** Quantitative RT-PCR analysis of mRNA levels of Glo-1 relative to hypoxanthine guanine phosphoribosyltransferase (HPRT), *n* = 5 for each condition. **b** Dimeric Glo-1 protein expression relative to Ponceau staining, *n* = 5 in each group. **c** Glo-1 enzymatic activity, *n* = 5 in each group. **d** Ponceau staining and Western blot as an illustrative example. Data are presented as median and interquartile range. **p = 0.0313

In IL-1β-stimulated chondrocytes, MitoTEMPO increased Glo-1 mRNA levels compared with the IL-1β condition alone (mean fold increase of 1.31 between IL-1β alone and IL-1β plus MitoTEMPO, *p* = 0.03) and thus restored the basal Glo-1 mRNA level (Fig. 6a), but the IL-1β effect was not prevented by MitoTEMPO.

MitoTEMPO increased Glo-1 mRNA expression, as well as protein expression (Fig. 6b), with IL-1β as well as without IL-1β stimulation. Indeed, the Glo-1 enzymatic activity decrease in IL-1β-stimulated chondrocytes was reversed by MitoTEMPO (mean fold increase of 1.27 between IL-1β alone and IL-1β plus MitoTEMPO, *p* = 0.03; Fig. 6c).

### Relationship between IL-1β-induced Glo-1 decrease and Nrf-2

**Fig. 7:**
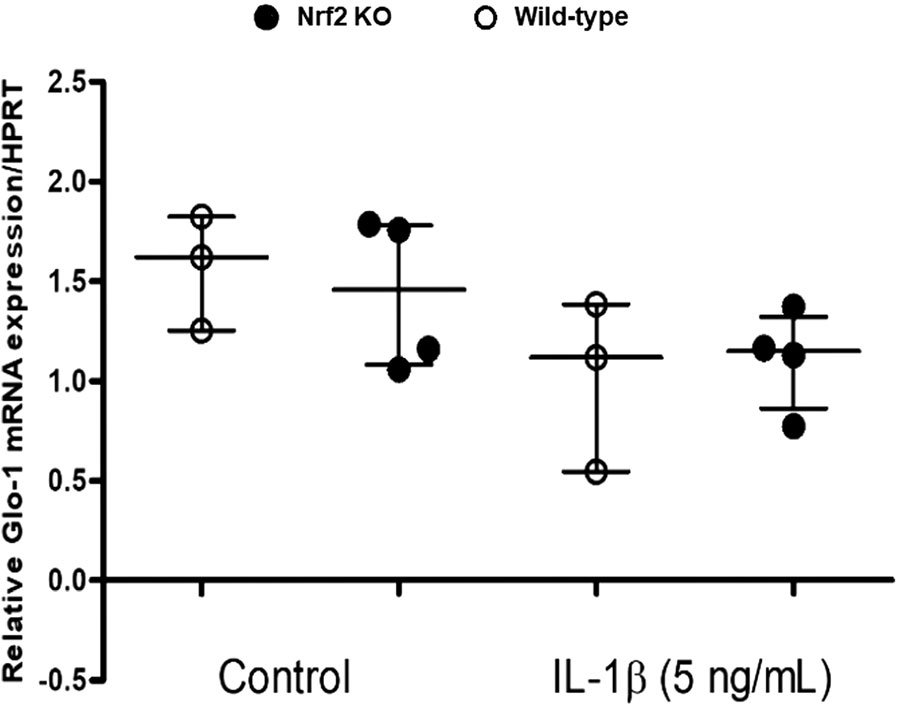
Involvement of nuclear factor-erythroid 2-related factor-2 (Nrf-2) in the downregulation of glyoxalase-1 (Glo-1) induced by interleukin-1β (IL-1β; 5 ng/mL). Cells were obtained from wild-type or Nrf-2^−/−^ mice. Quantitative RT-PCR analysis of mRNA levels of Glo-1 relative to hypoxanthine guanine phosphoribosyltransferase (HPRT), *n* = 3 for wild-type, *n* = 4 for Nrf-2^−/−^. Data are presented as median and interquartile. KO knockout

To further investigate the role of oxidative stress, we aimed to determine whether Nrf-2, the main transcription factor involved in the transcription of numerous antioxidant genes, plays a significant role in the Glo-1 decrease caused by IL-1β using articular chondrocytes extracted from Nrf-2^−/−^ mice. In Nrf-2^−/−^ chondrocytes, the basal mRNA expression of Glo-1 was not significantly different from that in wild-type chondrocytes (Fig. 7). With IL-1β stimulation, Glo-1 mRNA levels decreased similarly in Nrf-2^−/−^ and wild-type chondrocytes.

## Discussion

Here, we showed that, in contrast to other AGEs, CML accumulates in human OA cartilage with age, potentially due to IL-1β-induced impairment of physiological regulation of the detoxifying enzyme Glo-1. Moreover, in vitro, chondrocytes stimulated by IL-1β displayed decreased expression of Glo-1 (mRNA, protein levels, and enzymatic activity). This phenomenon was mediated by mitochondrial and NO-mediated oxidative stress.

The presence of CML in human healthy cartilage has been previously described by immunohistochemistry [7], but this is the first study to show CML accumulation in OA cartilage as a function of patient age. Interestingly, the positive correlation between the CML content in OA cartilage and patient age corroborated the results found for healthy human cartilage [7]. Diabetic patients were excluded from our experiments to avoid any confounding effects of diabetes on AGE levels. Indeed, in diabetes mellitus, AGEs are increased in several tissues [47, 48]. A specific CML effect on chondrocytes has not been studied, but synthetic mixtures of AGEs (mainly albumin modified by sugar, thus containing CML) has been reported to be deleterious to chondrocytes since they induce apoptosis, matrix metalloproteinase expression, and proinflammatory cytokine synthesis [11, 49].

Since AGEs are stable and irreversibly formed [19], AGE accumulation in cartilage with aging can be explained by the slow turnover of collagen in cartilage [7]. However, the absence of increasing Glo-1 activity with age may also be responsible for AGE accumulation since this enzyme removes the main CML precursor, glyoxal, and thus prevents the formation of CML [50]. This is the first time that Glo-1 expression has been explored in cartilage, despite the speculated role of the Glo enzymatic system in arthritis proposed several years ago [24]. With aging, Glo-1 enzymatic activity does not physiologically increase in human OA cartilage, which is consistent with results found in the brain and red blood cells [20]. Furthermore, cartilage samples showed an IL-1β-induced decrease in Glo-1 activity, suggesting that IL-1β-mediated inflammation may block the physiological regulation of Glo-1 [36]. Likewise, a vicious cycle may occur in aging-related OA: systemic inflammation (inflammaging) decreases Glo-1 expression, resulting in AGE accumulation. This AGE accumulation is then responsible for the local inflammation that perpetuates the decreased Glo-1 expression. In this study, contrary to Forsyth et al. [36], we did not see an increased sensitivity of old chondrocytes to IL-1β on Glo-1.

To better establish the relationship between inflammation/oxidative stress and CML accumulation in vivo, we should have been able to demonstrate the presence of an inflammatory activity (such as IL-1 or IL-6) in the OA cartilage and to measure the oxidative stress in this tissue to show a relation with Glo-1. However, some studies have already shown that IL-1β-mediated inflammation and AGE accumulation are two features of the OA cartilage [12] and have oxidative stress in common. Furthermore, Laiguillon et al. [37], using the same culture of murine chondrocytes as us, have shown that IL-1β induces several markers of oxidative stress. The link between oxidative stress and AGE has also been demonstrated [13]. This is why it did not seem useful to repeat these experiments.

We have previously shown that IL-1β induces several markers of oxidative stress in murine culture chondrocytes [37] and that AGE synthesis is associated with oxidative stress [37]. Thus, we aimed to inhibit NO synthase and mitochondrial ROS production by l-NAME and MitoTEMPO, respectively—two chemical compounds classically used to inhibit oxidative stress [51, 52]. Inhibition of NO synthase restored Glo-1 expression that had been downregulated by IL-1β. These results corroborate those reported by Miller et al. [53] who showed that increasing the intracellular concentration of NO decreased Glo-1 mRNA levels in pericytes and that NO decreased Glo-1 enzymatic activity. MitoTEMPO seemed to be less efficient than l-NAME in restoring the Glo-1 mRNA level. ROS, similar to NO, may have a dual effect by simultaneously stimulating Nrf-2, a transcription factor involved in the cellular response to oxidative stress [38, 39], and nuclear factor kappa B (NFκB), which can also directly downregulate Nrf-2 [54]. The Glo-1 promoter contains Nrf-2 and NFκB binding domains [55, 56]. In contrast to a previous study [39], we did not observe a relationship between basal Glo-1 mRNA expression and Nrf-2, with concordant results between experiments. Additionally, Nrf-2 was not involved in the inhibition of Glo-1 mRNA expression by IL-1β, but it likely involves oxidative stress (NO, mitochondrial ROS). Glo-1 downregulation may involve NFκB. In vivo, the decrease in Glo-1 may be more important than observed in vitro because AGEs favor NFkB nuclear translocation and expression [57].

Among the limitations of this study, Glo-2 has not been studied. Indeed, the glyoxalase system is made up of two enzymes (Glo-1 and Glo-2), and Glo-1 reduction of methylglyoxal into lactate is dependent on GSH and Glo-2. Glo-1 is the main and limiting enzyme of the system, and tools to measure Glo-2 activity are not available currently making it difficult to perform studies on this enzyme. An aged-related reduction in GSSG/GSH has also been shown [58]; however, we added GSH for the assessment of Glo-1 activity and thus our results are not influenced by the intracellular GSH concentration. In vivo, Glo-1 activity could be lower, in particular in older patients. Moreover, most explants are obtained from patients between 60 and 80 years, limiting the chance to find a correlation of the activity level with age. However, this patient age range is characteristic of OA patients who undergo total knee arthroplasty. We were unable to include normal human cartilage in this study. Indeed, human chondrocytes are precious and costly, and murine chondrocytes obtained by our protocol are appropriate [41].

## Conclusions

In conclusion, AGEs and especially CML accumulate with age in OA cartilage. This phenomenon may be explained by an impairment of the Glo-1 enzyme activity in the inflammatory environment. Glo-1 impairment induced by inflammation could involve an increase in oxidative stress in an inflammatory milieu. Likewise, Glo-1 stimulation has potential as a new therapeutic strategy for OA related to age.

## Additional file

Additional file 1: **Table S1.** Clinical characteristics of the 30 patients with knee osteoarthritis included in the study. **Figure S1.** Glo-1 staining in human OA cartilage. Glo-1 in red. The control is rabbit immunoglobulin, *n* = 3. (DOCX 107 kb)

## Abbreviations

AGE: Advanced glycation end-product
BMI: Body mass index
CEL: N_ε_-carboxyethyl-lysine
CML: N_ε_-carboxymethyl-lysine
DMEM: Dulbecco’s modified Eagle’s medium
Glo: Glyoxalase
Glu: Glutamine
GSH: Reduced glutathione
HPRT: Hypoxanthine guanine phosphoribosyltransferase
IL: Interleukin
LC-MS/MS: Liquid chromatography coupled with tandem mass spectrometry
MetS: Metabolic syndrome
MG-H1: Methylglyoxal-hydroimidazolone-1
NFκB: Nuclear factor kappa B
NO: Nitric oxide
Nrf-2: Nuclear factor-erythroid 2-related factor-2
OA: Osteoarthritis
P: Penicillin
RAGE: Receptor of advanced glycation end-product
ROS: Reactive oxygen species
S: Streptomycin
SASP: Senescence-associated secretory phenotype
TBST: Tris-buffered saline and Tween 20
