# Supplementary material for: Impairment of glyoxalase-1, an advanced glycation end-product detoxifying enzyme, induced by inflammation in age-related osteoarthritis
Source: Arthritis Res Ther. 2019 Jan 11;21:18. doi: 10.1186/s13075-018-1801-y (PMC6330409; doi:10.1186/s13075-018-1801-y)
Supplement: Supplementary file 1 — Table S1. Clinical characteristics of the 30 patients with knee osteoarthritis included in the study. Figure S1. Glo-1 staining in human OA cartilage. Glo-1 in red. The control is rabbit immunoglobulin, n = 3. (DOCX 107 kb) [file 13075_2018_1801_MOESM1_ESM.docx]

**Table S1.** Clinical characteristics of the 30 patients with knee osteoarthritis included in the study.

**Figure S1**. Glo-1 staining in human OA cartilage. Glo-1 in red. The control is rabbit immunoglobulin, n=3.

**Table S1**

| Clinical characteristics | AGE  (11) | Glo-1  (21) | p |
| --- | --- | --- | --- |
| Age (years)  Age range (years) | 67.4 ± 7.9  55 - 78 | 69.7 ± 9  49 - 87 | 0.6 |
| Women | 9 (82%) | 16 (76%) | 0.76 |
| BMI (kg/m²) | 29.7 ± 4.5 | 28.8 ± 5 | 0.91 |
| Obesity (BMI ≥30 kg/m²) | 3 (33%) | 8 (40%) | 0.5 |
| Diabetes mellitus | 0 | 0 |  |
| Hypertension | 8 (80%) | 10 (50%) | 0.39 |
| Dyslipidemia | 5 (50%) | 5 (25%) | 0.8 |
| Hyperuricemia | 0 | 0 |  |
| Smoker | 0 | 2 (10%) | 0.08 |
| Lequesne index | 10 ± 2.7 | 10.7 ± 3 | 0.18 |

The data are the mean ± standard deviation (SD) or n (%).

BMI: body mass index. AGE: advanced glycation end-products

Glo-1: glyoxalase-1

**Figure S1**

**
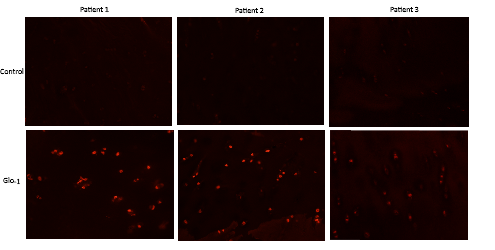
**
